# Protective effect of magnesium on renal function in STZ-induced diabetic rats

**DOI:** 10.1186/s40200-014-0084-3

**Published:** 2014-08-16

**Authors:** Mohammad Reza Parvizi, Mohsen Parviz, Seyed Mohammad Tavangar, Nepton Soltani, Mehri Kadkhodaee, Behjat Seifi, Yaser Azizi, Mansoor Keshavarz

**Affiliations:** Department of Physiology, School of Medicine, Tehran University of Medical Sciences, Tehran, Iran; Department of Pathology, School of Medicine, Tehran University of Medical Sciences, Tehran, Iran; Department of Physiology, School of Medicine, Hormozgan University of Medical Sciences, Bandar Abbas, Iran

**Keywords:** Diabetes mellitus type 1, Streptozocin, Diabetic nephropathy, Oxidative stress

## Abstract

**Background:**

Diabetic nephropathy is a serious complication of T1D (type one diabetes mellitus). Persistent hyperglycemia and subsequent hypomagnesemia is believed to develop kidney damage by activation of oxidative stress. We conducted this study to investigate the renoprotective effect of magnesium sulfate (MgSO_4_) on renal histopathology and oxidative stress in diabetic rats.

**Methods:**

The study included 70 male rats. The animals were divided into seven groups: control (CRL), control receiving MgSO_4_ (CRL + Mg1 & CRL + Mg8), diabetic (DM1 & DM8) and diabetic receiving MgSO_4_ (DM + Mg1 & DM + Mg8). Rats were given 20 mg/kg (i.p) Streptozocin (STZ) for 5 consecutive days in (MLD) multiple low doses to induce T1D. At day 10 treatment groups were received MgSO_4_ (10 g/l) in drinking water, for 1 or 8 weeks. The blood glucose, BUN and creatinine levels were measured. Renal tissue levels of malondialdehyde (MDA) were measured by thiobarbituric acid (TBA) method to evaluate the oxidative stress. Renal histopathology was done using H & E staining method.

**Results:**

Treatment with MgSO_4_ significantly decreased the blood glucose in DM + Mg1 and DM + Mg8 groups as compared with DM1 and DM8. Magnesium treatment also decreased serum BUN and tissue level of MDA significantly in both short and long term treatment. The body weight loss and kidney weight to body weight ratio was improved by MgSO_4_. Histological results showed there were no differences between DM and DM + Mg groups.

**Conclusion:**

Our findings showed that diabetic nephropathy is associated with high blood glucose level and oxidative stress (significant increase in MDA level). The renal dysfunction and oxidative stress can be improved by magnesium sulfate administration. It is suggested that protection against development of diabetic nephropathy by MgSO_4_ treatment involves changes in the blood glucose and oxidative stress.

## Introduction

Diabetic nephropathy is a major long-term complication of Type 1 diabetes mellitus (T1D) [1-3]. It develops in more than of 40% of patients in spite of glucose control [4]. Oxidative stress has been considered to be a pathogenic factor for diabetic nephropathy [5]. Hyperglycemia is believed to activate oxidative stress resulting in proteinuria, basement membrane thickening, expansion of the mesangium, decline in filtration and nephromegaly followed by glomerular sclerosis [2,6]. It is suggested that increased oxidative stress through reduction of plasma antioxidants and increased lipid peroxidation could intensify mesangial cells susceptibility to free radical injury [6,7]. Malondialdehyde (MDA) is a substance produced during polyunsaturated fatty acid peroxidation which has been detected in the serum of patients with T1D and correlate with the progression of disease [8-10]. MDA is known to have toxic influence on cell membrane structure. It can modulate signal transduction as well as modify proteins and DNA [11].

Magnesium (Mg) has received important attention for its potential in improving diabetes and its complications [12]. Mg deficiency has previously been proposed as a prominent factor leading to the pathogenesis of the diabetes complications [13]. Hypomagnesemia has been implicated in the development of diabetic complications due to enhanced renal Mg excretion [6,13,14]. The study of Prabodh suggested that hypomagnesemia may be related to development of diabetic nephropathy [15]. Hypomagnesemia was found to be related to weak glycemic control and increased incidence of nephropathy and retinopathy [16]. The study of *Srivastava* indicated that MgSO_4_ can improve oxidative stress by decreasing MDA generation in sodium-metavanadate induced lipid peroxidation [17]. However, the possibility that MgSO_4_ could exert beneficial effects in improving diabetic renal damage has not been previously investigated. It seems that Mg as magnesium sulfate (MgSO_4_) will be able to effectively limit oxidative stress. Thus this study was conducted to evaluate the renoprotective and antioxidant effects of oral magnesium administration on STZ- Induced diabetic rats in short and long term periods.

## Materials and methods

Male Wistar rats, weighing 200 ± 20 g were used. Animals were kept at a constant temperature of 22 ± 2°C with fixed 12:12-h light-dark cycle. Animals were divided into seven groups (n = 10): Control (CRL), control treated by magnesium sulfate for one week (CRL + Mg1), control treated by magnesium sulfate for 8 weeks (CRL + Mg8), untreated diabetic for one week (DM1), untreated diabetic for 8 weeks (DM8), diabetic treated by magnesium sulfate for one week (DM + Mg1) and diabetic treated by magnesium sulfate for 8 weeks (DM + Mg8). All the experiments were approved by the Ethics committee of Tehran University of Medical Sciences (Tehran, Iran).

### Diabetes induction

Diabetes were induced by intraperitoneal (i.p) injection of multiple low doses (20 mg/kg) of STZ (Sigma-Aldrich Inc., USA) for 5 consecutive days [18]. In 10^th^ days, the blood glucose levels were determined using a glucometer (ACCU-CHEK Active, Germany) and animals with blood glucose levels above 250 mg/dl were considered to be diabetic [19]. Control rats were injected with the same volume of vehicle. Mg-treated rats were received 10 g/l of MgSO_4_ added to the drinking water from 10^th^ days [20]. Untreated groups of STZ- diabetic and control rats were given drinking water over the same period.

### Blood sampling and biochemical assay

Blood samples were taken for glucose, creatinine, BUN, calcium and Mg levels measurements using a kit (Zistshimi, Tehran, Iran) and spectrophotometer (UV 3100, Shimadzu). Blood samples were centrifuged at 2000 g for 10 minutes at 4°C, and serums were used for biochemical assay. All analysis was performed in accordance with the instructions provided by the manufacturers. Serum creatinine concentration was determined using Jaffe method [21] and BUN was determined using UV method by autoanalyzer (BT 3000).

### Histological analysis

At the end of experiment, rats were euthanized with high-dose of ketamine. The right kidney of the animals in all groups were identified, resected, dried by tissue papers and weighed by digital scale on Sartorius balance to determine the change in the weight of organs with respect to their body weights. A change in kidney size was estimated by division the weight of the right kidney to total body weight [1]. The kidney was removed from each rat, put into 10% formalin and embedded in paraffin. Each sample was then cut into 5-*μ*m sections with a microtome and deparaffined with xylene. Then sections are subjected to standard hematoxylin/eosin staining. The sections were observed under a light microscope at magnifications of 400x [22-24]. Determination of MDA in lipid peroxidation study was performed by thiobarbituric acid (TBA) method [25]. Briefly, the renal tissue was mixed with 2 volumes of 10% trichloroacetic acid (TCA) for protein precipitating. After centrifugation of the precipitate, supernatant is separated and reacted with TBA in boiling water for 10 min followed by cooling. The concentration of MDA was measured at 532 *n*m [26,27].

### Statistical analysis

Results were expressed as mean ± SEM. Differences among groups were evaluated by one- way analysis of variance (ANOVA) with Tukey post-hoc test. Statistical significance was achieved if the p < 0.05.

## Results

### Effects of magnesium sulfate administration on blood glucose

**Figure 1:**
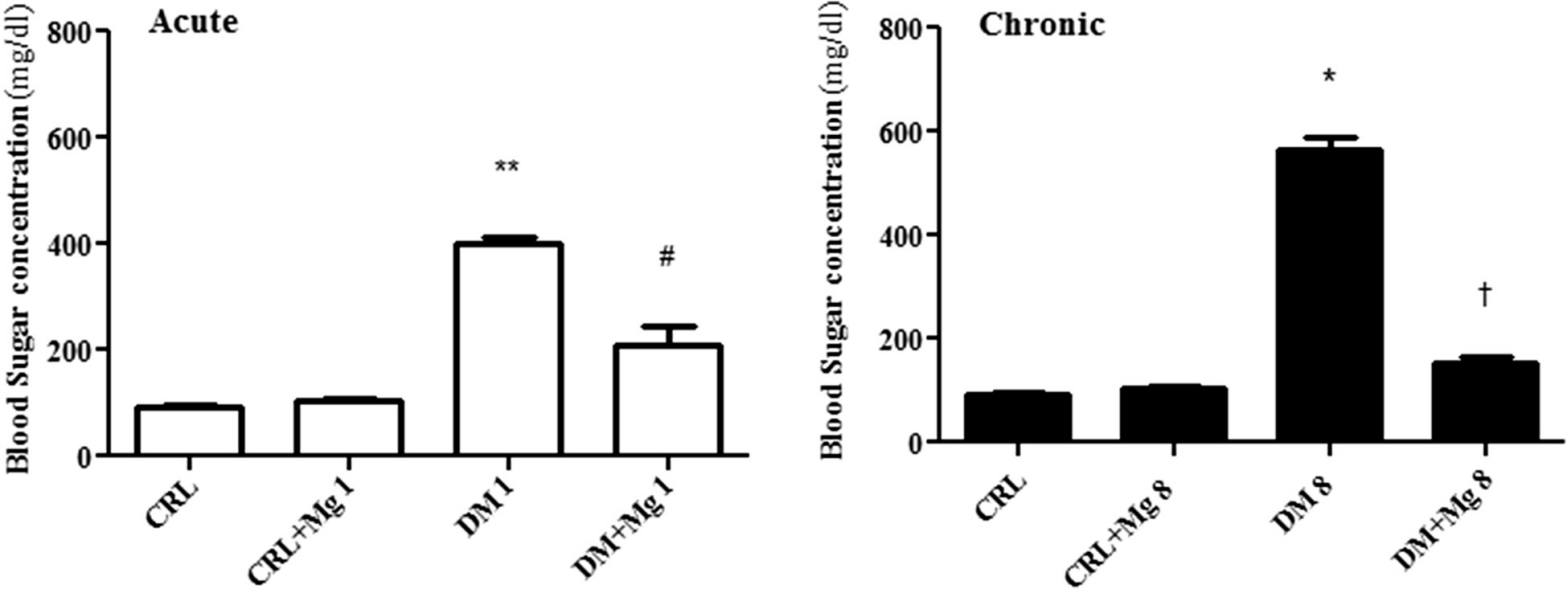
**Mean blood glucose level in CRL (control), CRL + Mg (control rats treated with MgSO**_**4**_ **), DM (diabetic) and DM + Mg (diabetic rats treated with MgSO**_**4**_ **) in one and 8 weeks after diabetes induction by STZ (** ***n*** **= 10).** ** p < 0.01 vs CRL and CRL + Mg1, # p < 0.05 vs DM1, * p < 0.01 vs CRL and CRL + Mg8, † p < 0.001 vs DM8.

Before the experiment, there were no significant differences between blood glucose of groups (93.1 ± 5.1 vs 98.1 ± 3.1). At 10^th^ days of study, blood glucose of diabetic animals significantly increased to opposite the non-diabetic ones (398.9 ± 15.3 vs 103.0 ± 6.2) [F_(3,36)_ = 80.67], (p < 0.001). Treatment with MgSO_4_ significantly (p < 0.01) decreased the blood glucose in DM + Mg1 (230.4 ± 5.8 mg/dl) and DM + Mg8 (153.5 ± 12.6 mg/dl) [F_(3,36)_ = 292.3], (p < 0.001) in comparison with DM1and DM8 respectively (Figure 1).

### Change in body weight and kidney weight/body weight (KW/BW) ratio

**Figure 2:**
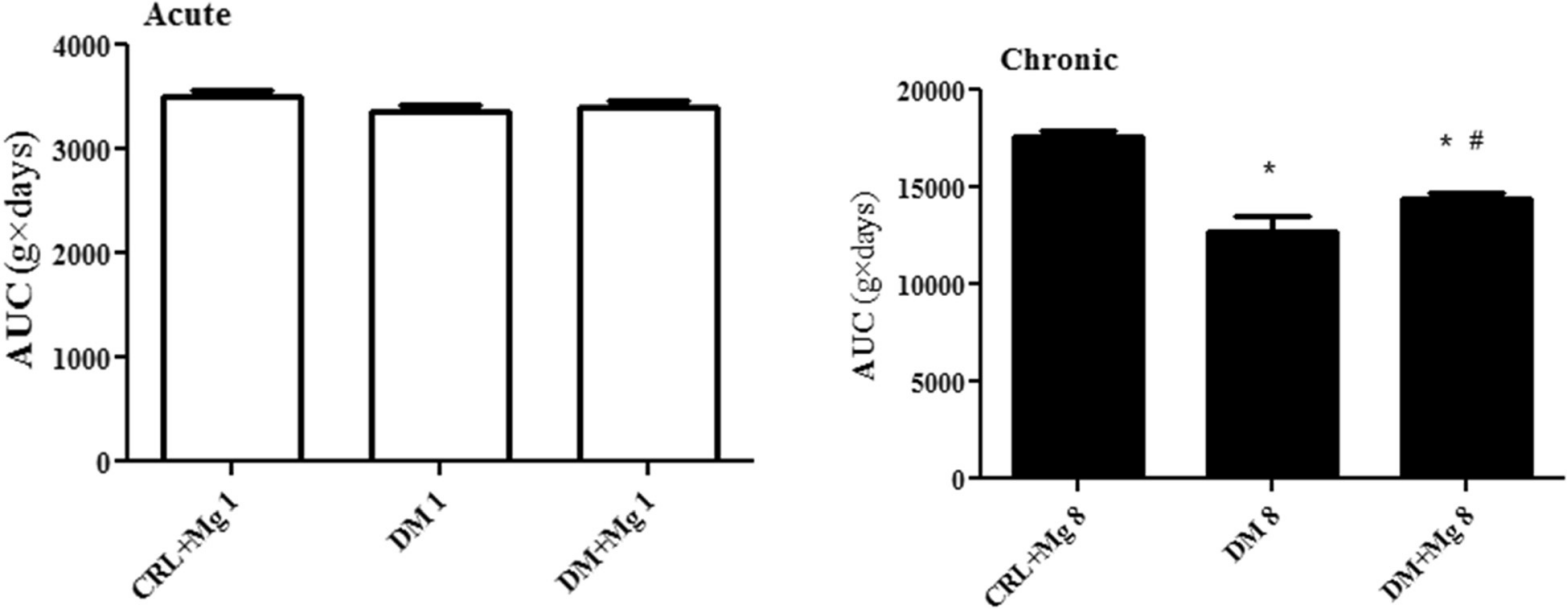
**Effect of magnesium sulfate treatment on body weight changes.** CRL + Mg: control rats received magnesium sulfate, DM: untreated diabetic rats, and DM + Mg: diabetic treated with magnesium sulfate (10 g/l added in water) in one and 8 weeks (n = 10). AUC: area under curves of body weight changes during one and eight weeks (g × days) were expressed and compared. Data are presented as mean ± SE. * p < 0.05, *** p < 0.001 vs, CRL + Mg8, # p < 0.05 vs DM8.

**Figure 3:**
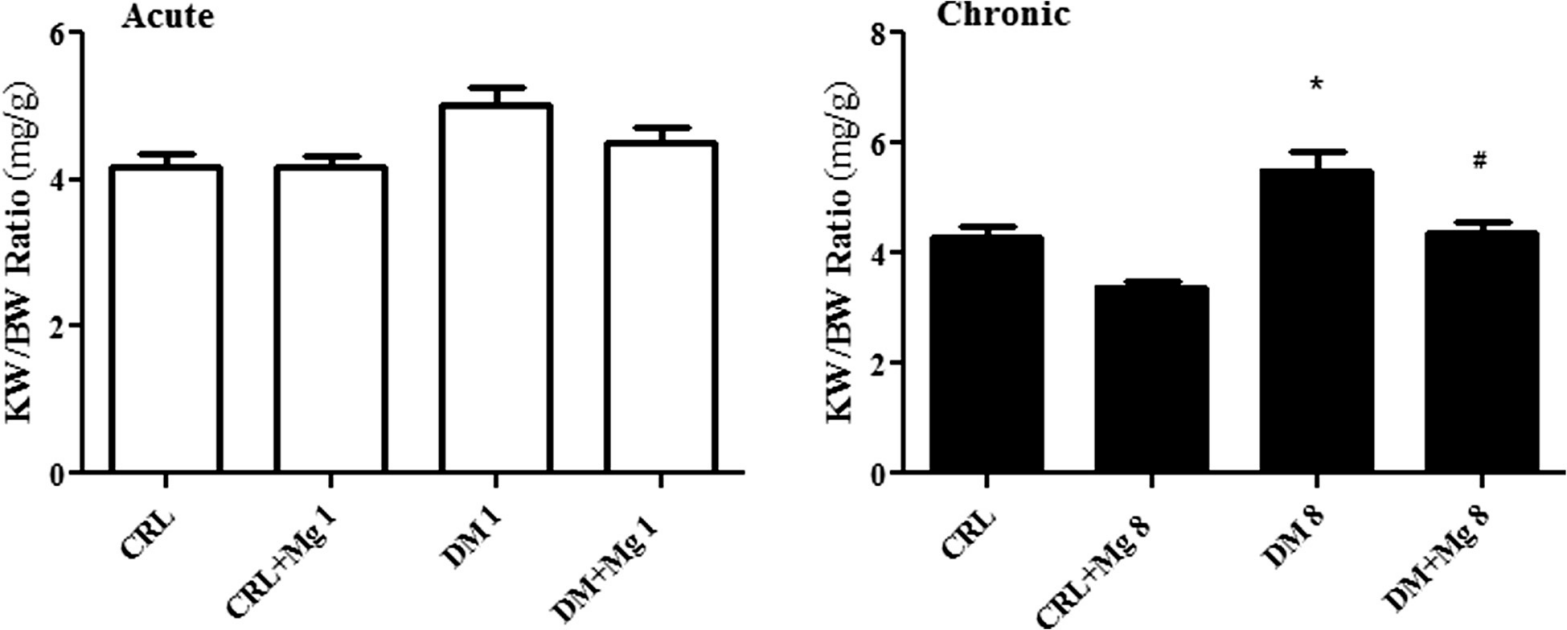
**Effect of magnesium sulfate treatment on kidney weight/body weight ratio in control rats (CRL), control rats treated with magnesium sulfate (CRL + Mg), Untreated diabetic rats (DM) and diabetic rats treated with magnesium sulfate (DM + Mg) (10 g/l added in water) in 1 and 8 weeks (n = 10).** Data are presented as mean ± SE. * p < 0.05 vs. CRL, CRL + Mg8, # p < 0.05 vs DM8.

There was significant reduction in body weight of DM1 compared to CRL + Mg1 group (209.4 ± 6.3 g vs. 228.5 ± 4.2 g) [F_(2,27)_ = 1.696] as well as DM8 compared with CRL + Mg8 group (194.7 ± 8.8 g vs. 230.8 ± 13.1 g) [F_(2,27)_ = 32.53] (p < 0.0001). Magnesium treatment prevented body weight loss in DM + Mg1 and DM + Mg8 compared with DM1 and DM8 significantly (219.1 ± 11.6 g vs. 194.7 ± 8.8 g) (p < 0.01) (Figure 2). In addition, diabetic rats had an increased kidney weight/body weight ratio, a marker for the body weight loss and renal size [28]. This ratio was significantly improved by treatment with MgSO_4_ in DM + Mg8 groups (Figure 3).

### Effects of magnesium sulfate administration on plasma level of Mg^2+^

**Figure 4:**
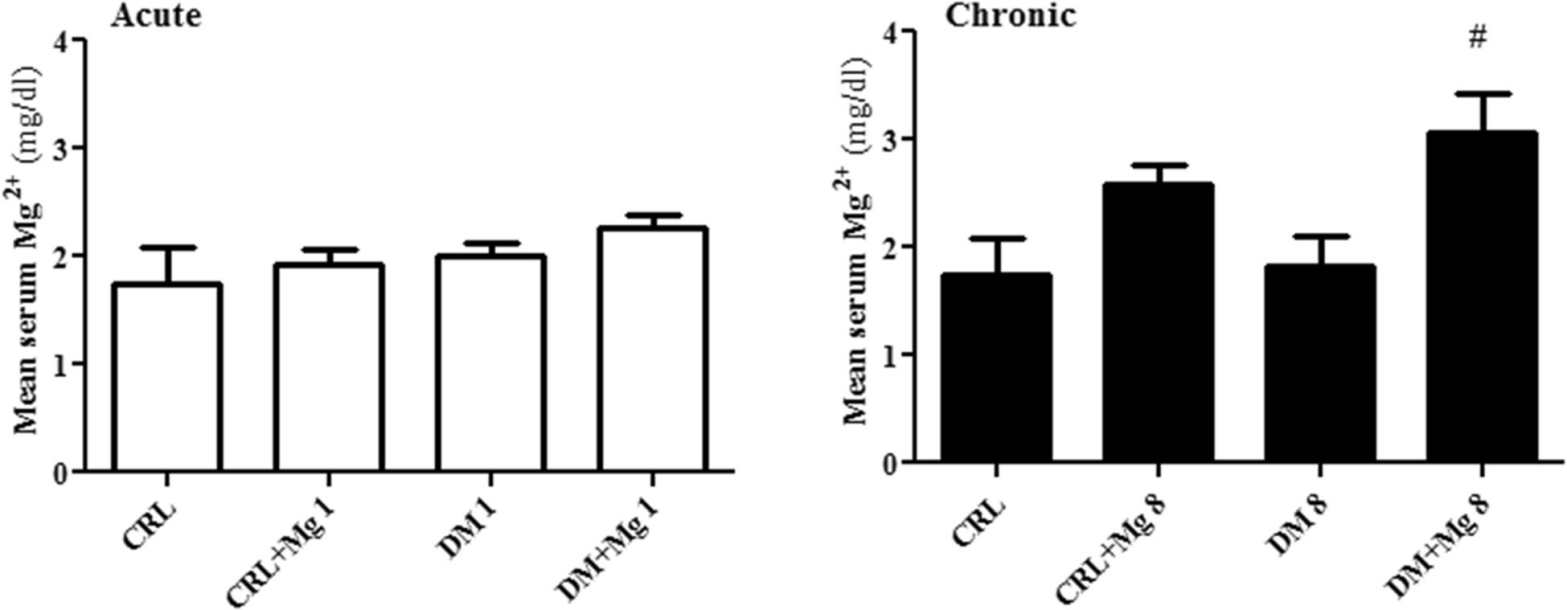
**Serum level of magnesium (Mg** ^**2+**^ **) in CRL (control), CRL + Mg (control rats treated with MgSO**_**4**_ **), DM (diabetic) and DM + Mg (diabetic rats treated with MgSO**_**4**_ **) in 1 and 8 weeks after diabetes induction by STZ (** ***n*** **= 10).** Data are presented as mean ± SE. # p < 0.05 vs DM8.

Plasma magnesium level in both DM1 and DM + Mg1 groups didn’t show any changes in comparison with control (2.0 ± 0.13 and 2.26 ± 0.12 mg/dl) [F_(3,36)_ = 0.9309]. 8 weeks after diabetes induction by STZ, magnesium level of plasma decreased in DM8 as compared to CRL + Mg8 groups. MgSO_4_ treatment could increase the plasma level of magnesium significantly in DM + Mg8 as compared to DM8 groups (3.33 ± 0.3 vs 1.74 ± 0.4 mg/dl) [F_(3,36)_ = 3.812] p < 0.05 (Figure 4).

### Effects of magnesium sulfate administration on plasma level of BUN and creatinine

**Figure 5:**
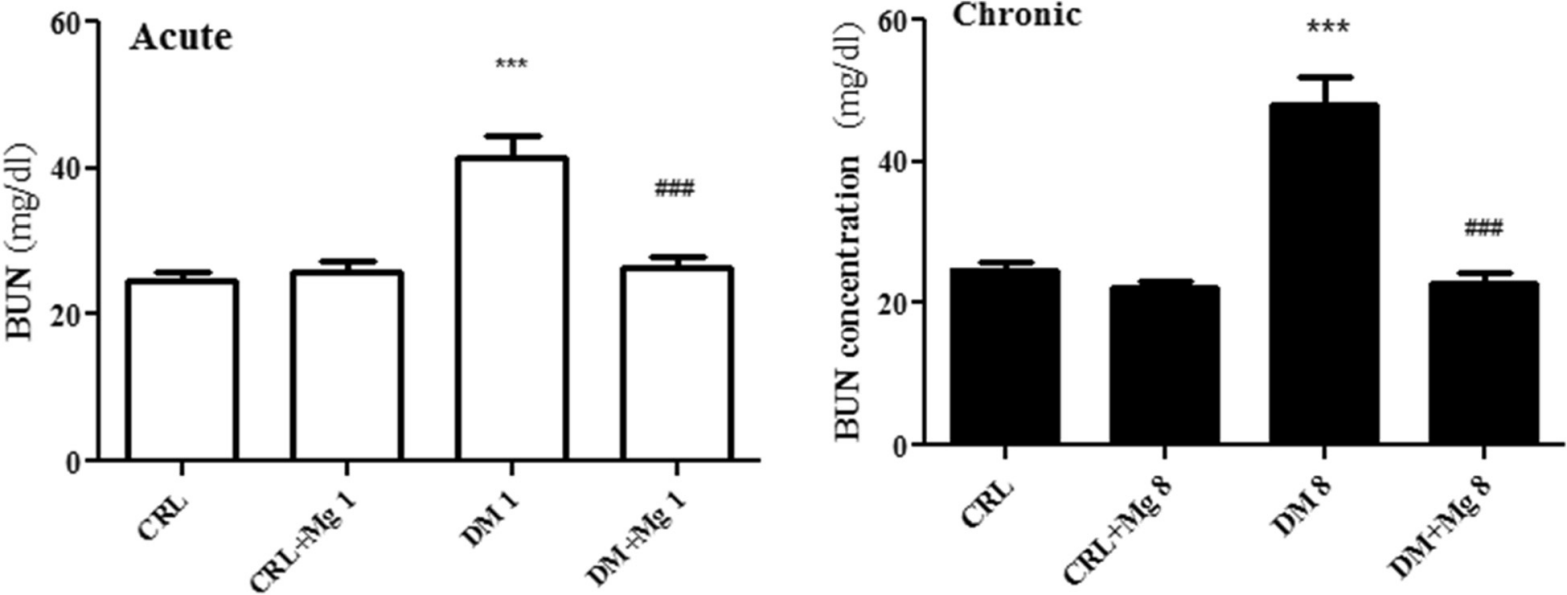
**Serum level of BUN in CRL (control), CRL + Mg (control rats treated with MgSO**_**4**_ **), DM (diabetic) and DM + Mg (diabetic rats treated with MgSO**_**4**_ **) in one and 8 weeks after diabetes induction by STZ (** ***n*** **= 10).** Data are presented as mean ± SE. *** p < 0.001 vs. CRL, CRL + Mg, ### p < 0.001 vs DM.

Our results showed that STZ- induced diabetes caused a significant increase in serum BUN level in DM1 (41.37 ± 2.95 mg/dl) and DM8 (48.10 ± 3.79 mg/dl) compared with CRL, CRL + Mg1 and CRL + Mg8 (24.77 ± 1.05, 25.83 ± 1.5 and 22.3 ± .66 mg/dl) respectively [F_(3,36)_ = 17.53] (p < 0.05). Following treatment with magnesium sulfate serum BUN levels decreased in DM + Mg1 (26.3 ± 1.49 mg/dl) and DM + Mg8 (22.75 ± 1.68 mg/dl) groups, in comparison with DM1 (41.37 ± 2.95) and DM8 (48.10 ± 3.79) respectively [F_(3,36)_ = 28.12] (p < 0.05) (Figure 5).

**Figure 6:**
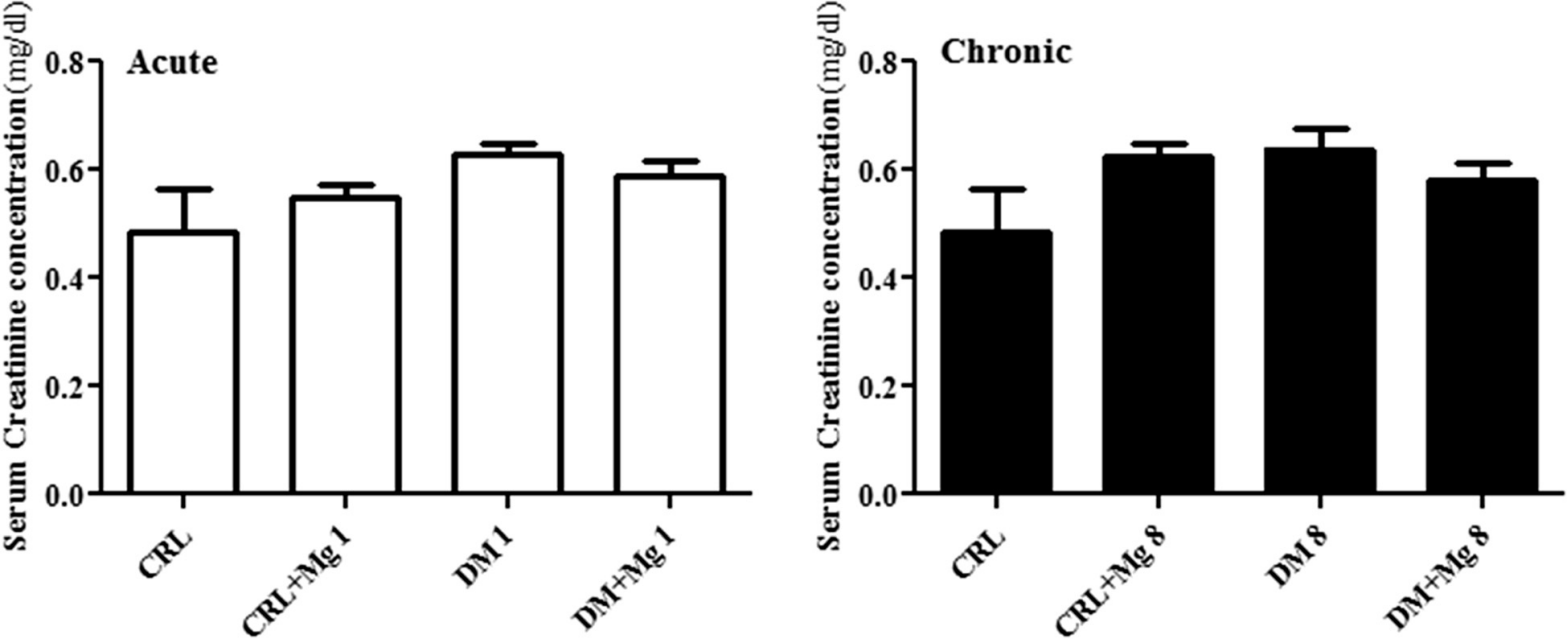
**Serum level of Creainine in CRL (control), CRL + Mg (control rats treated with MgSO**_**4**_ **), DM (diabetic) and DM + Mg (diabetic rats treated with MgSO**_**4**_ **) in one and 8 weeks after diabetes induction by STZ (n = 10).** Data are presented as mean ± SE.

After diabetes induction by STZ, a mild increase in creatinine level showed in 1 and 8 week groups (0.62 ± 0.018 and 0.64 ± 0.13 ± 0.03 mg/dl) [F_(3,36)_ = 1.971] respectively. There were no significant differences in serum creatinine level between DM and DM + Mg in both weeks groups (0.59 ± 0.02 and 0.58 ± 0.03 mg/dl) [F_(3,36)_ = 2.368] respectively (Figure 6).

### Effects of magnesium sulfate treatment on MDA level in renal tissue

**Figure 7:**
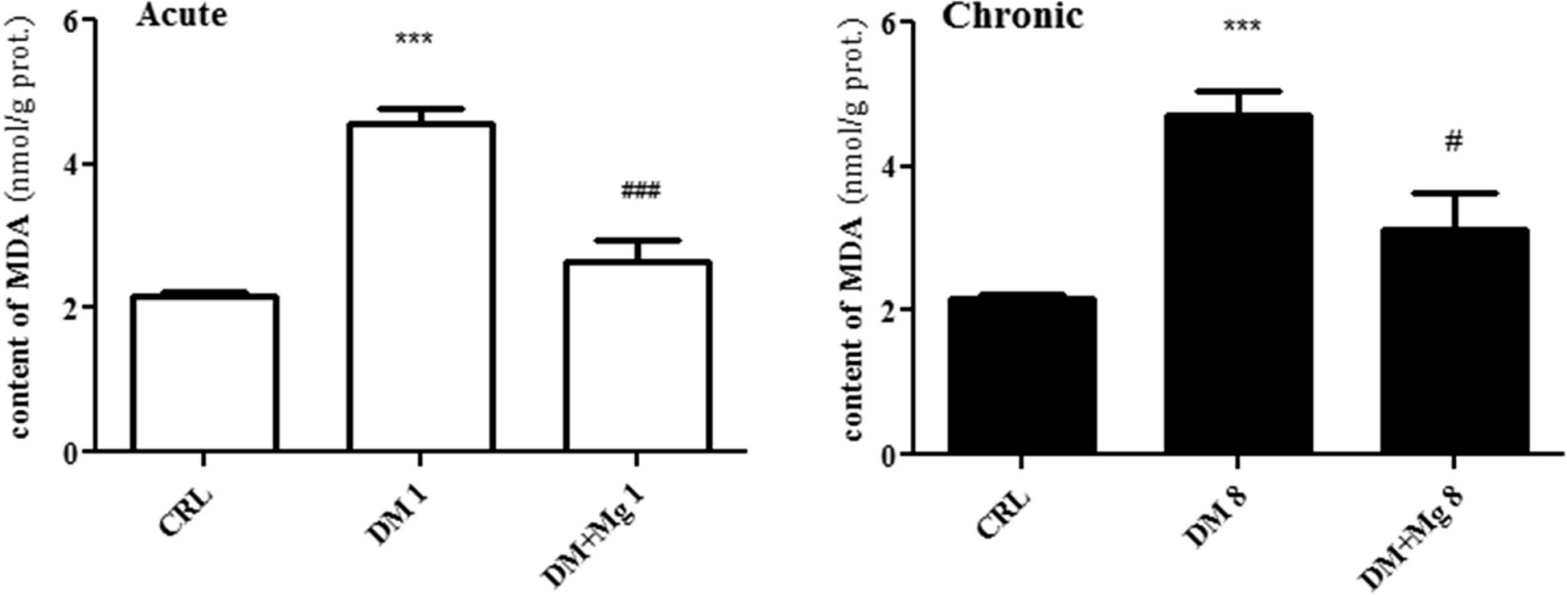
**Kidney tissue level of MDA in CRL (control), and DM + Mg (diabetic rats treated with MgSO**_**4**_ **) in 1 and 8 weeks after diabetes induction by STZ (** ***n*** **= 10).** Data are presented as mean ± SE. *** P < 0.001 vs CRL, ### P < 0.001 vs DM1, # P < 0.05 vs DM8.

MDA was used as a marker of oxidative stress [29]. The renal content of MDA was higher levels significantly in DM1 and DM8 (4.55 ± .21 and 4.72 ± .31 nmol/g protein) respectively in comparison with CRL (2.17 ± .05 nmol/g protein) [F_(2,27)_ = 34.94]. Magnesium treatment attenuate renal content of MDA in DM + Mg1 and DM + Mg8 (2.64 ± .31 and 3.12 ± .51 nmol/g protein) [F_(2,27)_ = 12.13] significantly compared to DM1 and DM8 respectively. However, there was no difference in renal content of MDA between DM + Mg1 and DM + Mg8 (Figure 7).

### Histological study

**Figure 8:**
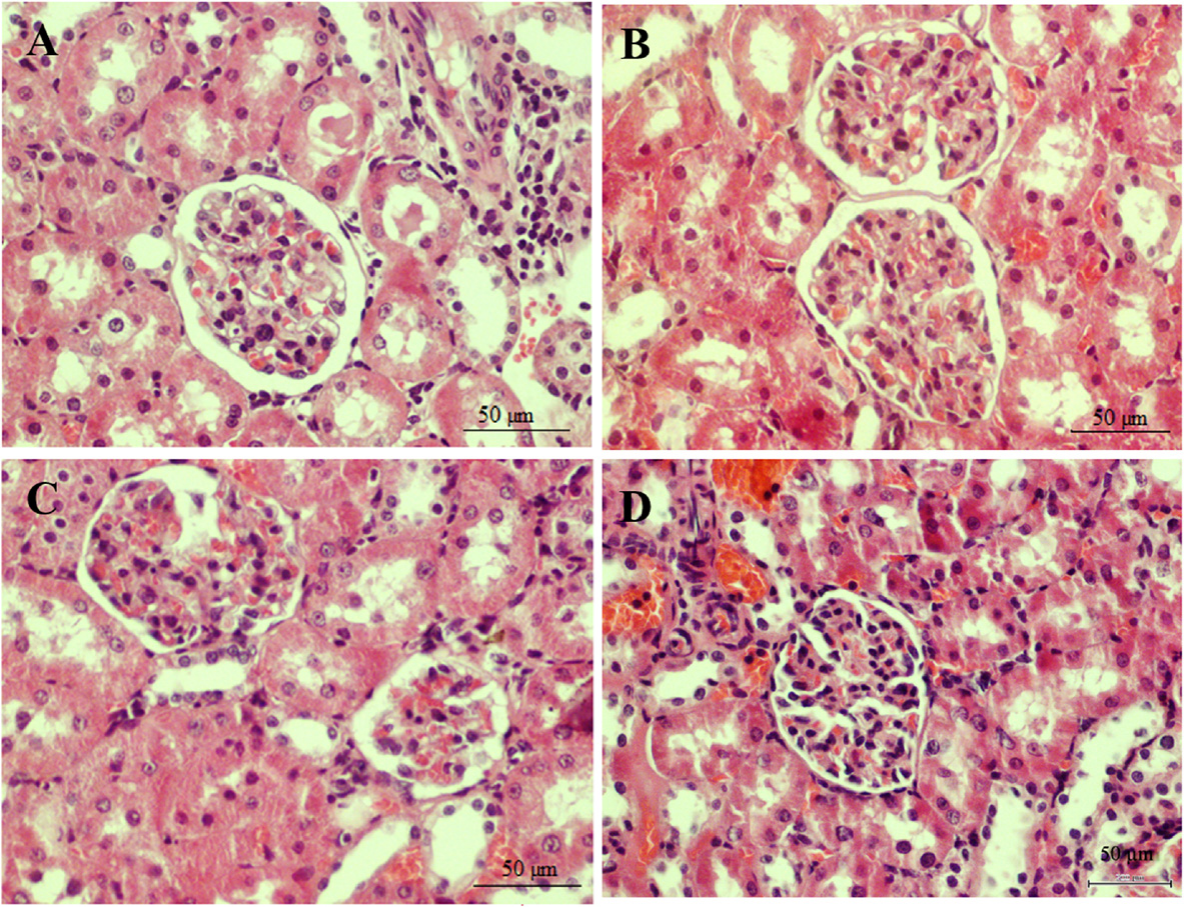
**The images of kidney tissues (magnification x 400) in untreated diabetic rats (A & C) and diabetic treated by magnesium (B & D) in 1 and 8 weeks after diabetes induction by STZ. A**: DM1, **B**: DM + Mg1, **C**: DM8, **D**: DM + Mg8.

Results of the renal tissue staining showed that the glomeruli of the control and diabetic groups were morphologically normal and that the treatment of the diabetic groups in 1 and 8 week did not have a clear effect on the structure of the glomeruli (Figure 8).

## Discussion

Diabetes mellitus can cause serious health problems including macrovascular and microvascular complications such as kidney failure, heart disease, stroke and etc., which affects the function of many organs [4]. One of them is injuries to the kidney tissue that results in renal dysfunction [30]. This study was designed to evaluate the renal complication of T1D in short and long term and probable protective effects of MgSO_4_. We used the multiple low doses of STZ to induce T1D in rats according to previous reports [18,31]. Eight weeks after STZ-diabetes induction, some indexes of renal damage such as increasing BUN and elevation of renal MDA were appeared. Magnesium treatment could reverse renal function (decreasing BUN), decreasing MDA and improving hyperglycemia and kidney weight respect to body weight in diabetic rats.

Previous studies has been proved that treatment with MgSO_4_ have beneficial effects on diabetes. It could improve some of diabetic complications such as hypertension [19,32], thermal hyperalgesia and structure of pancreatic islets [20,33]. Also MgSO_4_ improves function of endothelium and restores hemodynamic and tubular function in postischemic rats [34]. With respect of soltani study [20], we used magnesium supplementation (10 g/l) via drinking water to diabetic rats from 10^th^ days after diabetes induction for short time (one week) or long time (eight weeks).

Our results showed that consumption of MgSO_4_ in diabetic rats reversed the high blood glucose level. However, MgSO_4_ couldn’t return it to that control levels in the treated diabetic rats. Magnesium plays an important role in the carbohydrate metabolism regulation [35]. We have been previously showed that treatment by MgSO_4_ could reduce the blood glucose in diabetic rats [20]. The effect of MgSO_4_ on blood sugar levels may be related to the fact that it is a crucial cofactor for glucose transport and various enzymes involved in carbohydrate metabolism [36]. It has also been reported that oral MgSO_4_ consumption in male obese rats improved glucose tolerance and delaying the progression of diabetes [37]. It is supposed that blood glucose levels can be decreased by Mg via increasing GLUT_4_ mRNA expression in diabetic rats independent to insulin secretion [38]. *Huang* et al. showed that MgSO_4_ increases the expression of GLUT_3_ in the cortex and hippocampus of gerbil brains [39].

We showed that after one week MgSO_4_ treatment (in drinking water), the plasma Mg levels in both DM1 and DM + Mg1 groups didn’t show any significant changes in comparison with control group. In previous work, *Soltani* et al., showed that a significant reduction in Mg level in acute diabetic groups [32]. These discrepancy may be due to the dose of STZ and the model of diabetes induction. Because in this study we used MLD injection of STZ, but in the *Soltani* et al., study they used single high dose of STZ for diabetes induction [20]. In other hand, after few days following diabetes induction, rats showed polydipsia and polyuria along with diarrhea (in DM + Mg groups). Actually, in long term diabetes, ingestion of water and meal, reached near to control rats. It may be due to adaptation.

High Magnesium (as MgSO_4_) ingestion has been reported to induce diarrhea. In short term diabetes, diarrhea and polyuria along with polydipsia can cause non-significant changes in magnesium level [39,40].

Our results showed that Mg administration to DM + Mg8, after eight weeks, could decrease the level of renal function markers such as BUN and creatinine in comparison to DM8. An elevation of BUN in DM8 groups may reflect decreasing in glomerular filtration rate (GFR) or it is probably due to increased degradation of proteins [41]. *Avram* et al., showed that there is a negative correlation between renal size and serum creatinine level [42]. So in our study, mildly elevation of creatinine may be associated with renomegaly as shown in this study.

Increasing BUN and plasma creatinine, has been reported as waste products of metabolism following kidney injury and they has known biomarkers of kidney degeneration [43]. In this study, the elevation of plasma BUN with hyperglycemia can be proposed as indicator for renal dysfunction [44]. Our data showed no significant changes in the creatinine level in diabetic animals versus CRL group. Increase in creatinine level occurs at the end stage of renal disease, and this is accompanied by histological alterations [45]. *Kim* et al., suggested that plasma BUN and creatinine is often measures together, but former is more sensitive marker for kidney injury [46].

Histological study also showed that there were no profound changes among the experimental groups. Eight weeks after induction of diabetes in DM8 groups’ tissue didn’t changes histologically. It seems that it requires long time to cause an end stage renal disease with changes in creatinine level and histological changes. In previous study, it has been shown that it take about 15 weeks to see profound histological changes [47]. So it may be due to insufficient time that we didn’t see this changes.

Diabetes induction resulted in an increase in kidney MDA, which is an indicator of oxidative stress. *Israa* et al., revealed that enhancement of the oxidative stress as indicated by high MDA level is due to increase in blood glucose [48]. Metabolically, diabetes is characterized by diminished glucose utilization and increased lipid peroxidation, resulting in accumulation of MDA in renal tissue. The elevated level of MDA may be due to the poor antioxidant capacity of mesangial cells as a result of low glutathione (GSH) levels [49]. Evidences showed that in patients with T2D, the higher levels of lipid peroxidation and hypomagnesaemia are often reported [50]. Free radicals in DM cause peroxidative breakdown of phospholipids that lead to accumulation of MDA [51]. *Agnieszka* et al., have been shown that magnesium intake for 18 weeks could prevent lipid peroxidation stimulated by vanadium in hepatic tissue [52]. Thus, from our results it is seemed that magnesium may show an antioxidant activity through preventing lipid peroxidation in renal tissue that is indicated by a decrease in kidney MDA level. *Ribeiro* et al., demonstrated a negative correlation between magnesium and glucose levels as well as between magnesium and oxidative stress [3]. Magnesium deficiency enhances oxidative stress by increased production of free radicals and decreased of antioxidant defenses [53]. In this regard, histological alterations can be due to mildly proinflammatory invasion to renal tissues.

Hyperglycemia induces diabetic nephropathy via several biochemical pathways. Several mechanisms have revealed to depict the adverse effect of hyperglycemia, including protein kinase C (PKC) [48], mitogen-activated protein kinase (MAPK), polyol pathway, advanced glycation end products (AGE), aldose reductase activation [54] and oxidative stress [55]. In the other hand, it has been reported that hypomagnesemia is a novel predictor of end stage renal disease (ESRD) in patients with type 2 diabetic nephropathy [14]. So it is suggested that oxidative stress causing damage to mesangial cells and magnesium could reduce oxidative damages to mesangial cells as indicated by decreasing MDA production.

In addition, we assessed the effects of magnesium administration on the body weight and kidney weight to body weight ratio in diabetic rats. We found that induction of diabetes caused body weight loss. Decrease in body weight following STZ-induced diabetes notably in eight weeks groups, may be due to degradation of proteins as indicated by raising serum BUN and creatinine levels. Area under curve showed that in DM + Mg groups, Mg treatment caused body weight gain, especially in DM + Mg8 groups.

Also our results showed that KW/BW ratio was significantly elevated in untreated diabetic rats and decreased in DM + Mg groups. KW/BW ratio is a marker of renomegaly [56], its increment is an indicator of glumerolar expansion due to diabetes. Several investigators have reported that KW/BW increases in DM animals [56]. *Renet* et al., showed that marked increase in the KW/BW, tubulointerstitial fibrosis and fibronectin in STZ-induced diabetic rats from 8 to 16 weeks [57]. *Arozal* et al., oxidative stress is associated with development of hypertrophy in diabetes [58]. *Sharma* et al., revealed that renal hypertrophy in T1D was related to overexpression of TGF-β1 in the glomerular mesangial cells [59]. Our study further strengthens the notion that attenuation of kidney weight by magnesium, suggesting that Mg has the ability to protect the kidneys from oxidative injury. It may reverse kidney hypertrophy in STZ-diabetic rats.

Limitations of this study was to evaluate more precisely the oxidant and antioxidant markers for achieving better results, because of limitation in financial support. We haven’t access to electron microscope for more precise evaluation of histological changes.

## Conclusion

The current study determined that the increase in blood glucose level is related to the development of oxidative stress as indicated by high MDA level. Control of hyperglycemia by Mg supplementation can decreases oxidative damages as indicated by MDA and improves renal dysfunction via lowering of BUN and creatinine. However, further studies are required to clear the precise mechanism(s) involving for the protective effect of MgSO_4_ against the diabetic nephropathy in experimental conditions.
